# Coccobacteria in Purulent Otorrhœa

**Published:** 1882-02-20

**Authors:** C. Lambert

**Affiliations:** Goshen, Indiana


### COCCOBACTERIA IN PURULENT OTORRHŒA.

BY C. LAMBERT, M.D., OF GOSHEN, IND.

**A Paper read before the Elkhart Co. Medical Association, at their October Meeting.**

MR. PRESIDENT AND GENTLEMEN OF THE SOCIETY:—I would like to call your attention to a subject of vital importance in the treatment of purulent otorrhœa, and point ont what, in my estimation, is and has been the cause of so many failures in arresting the breaking down and loss of the delicate organisms of the ears affected with this oppressive and destructive disease.

The chief cause of this putrid decomposition has been generally overlooked. I allude to the microscopic coccobacteria present in the pus, and the therapeutical indications furnished by their presence.

I design, with your permission, at our next meeting in December, to present slips for microscopic exhibition, and continue my remarks on this same subject.

Coccobacteria septica belongs properly to the domain of general bactorio-pathology, and I need not speak of this in particular, but call your attention to the recent investigations by eminent scientists and patient investigators, who have perfectly demonstrated the presence of these microphytes in the fetid ear discharge, and given us the cue for more rational therapeutics in these cases. It

I hope, will be granted me to quote a few lines from Billroth and Lowenberg, as the very latest we have on this subject.

"Lowenberg has, since June, 1880, carefully subjected to examination the products of secretion of the affected ears of all the patients coming under his care, in order to enable him to study the nature of the respective mycrophytes. The examination consisted, on the one hand, in the microscopic study of the pus (with all its accompanying detritus), obtained by syringing or otherwise; on the other hand, in attempts at cultivation, partly in the above mentioned media, and partly in boiled neutralized urine."

The experiments were obtained with all the precautions necessary and indispensable in such cases. The experiments demonstrated that in all these cases we had to deal with the ordinary organisms of decomposition.

"In all cases of otorrhœa where the cleansing is not done with the greatest care, and by the aid of suitable apparatus, the pus contains great numbers of micrococci. If, in consequence of persistent neglect, the secretion is allowed to become offensive, the micro-organisms swarm in incredible quantities."

"In this connection, we can state with decided emphasis that, contrary to the belief in competent circles, the pus secreted in simple purulent otorrhœa, in its fresh state, is as little offensive as that from other diseased mucous membranes, simply on account of the absence of any cause therefor. Fetor, then, according to their present opinion, points to stagnation and a high degree of decomposition dependent thereon, the existence of which is proved by the presence of micrococci.

"The most abundant secretion or multiplication of micrococi is found in those cases that have been treated with emollients, particularly with cataplasms These coccobacteria find all the aliments necessary to their growth and multiplication in the fetid ear discharges. If cataplasms increase the moisture and heat, and contribute additional organic material, we have an actual hot-house culture of bacteria.

"This condition explains the fact, well known to otologists, that, after the prolonged employment of cataplasms, almost interminable purulent processes in the ear often remain behind. Under this unintentional artificial cultivation, the putrefactive organisms reach a high degree of development, and in their turn keep up the prolonged suppuration. By a similar development and furtherance of micrococci, though of a special character, we may interpret the fact that after continued application of poultices, outbreaks of furuncles may be incited in any part of the body."

Recognizing the presence of coccobacteria in nearly all these cases, and being satisfied of their presence, I will offer some observations from recent practice, and suggest a course of treatment that has been very satisfactory in my hands. All of us are aware of the difficulty of treating properly these cases of perforation of the tympanum and chronic purulent catarrh of the middle ear, and I will reieterate by saying that the proper instruments are necessary: speculum, mirror, probes, syringes and absorbent cotton, etc., etc., and antiseptic remedies. The ear should be inspected every day, and carefully cleansed. I rely upon the syringe and absorbent cotton, and the appearance from day to day of the diseased parts is to dictate all the changes, if any, in the local treatment necessary.

I claim that no physician can treat successfully these cases as out-door cases. They must be surrounded with all care and circumspection; diet nutritious, and put, if necessary, upon alteratives and tonics, and cease entirely being exposed to the causes that originally produced the diseased condition; then we have a fair chance at the case. It may be a few days, or a few weeks, before we can get all the accumulations removed, so that when a remedy in liquid state is put into the meatus it can readily be forced through the middle ear and Eustchian tube. This is a *sine qua non* in the successful management of even the simplest case. The time need not exceed, upon an average, more than from two to four weeks, even in the worst cases presenting. I cleanse the ear with a weak solution of carbolic acid, then generally mop out all the pus and detritus, and then resort to the Eustachian catheter, and find that the battle is fairly won when air can be made to pass through all the channels; then, and only then, are medications indicated. As to the particular ingredients of said washes, they are many; some say this is best, some that, I avoid nitrate of silver and sul. cupri almost entirely, and depend upon mild astringent solutions of sul. zinc, sul. morphia, alum, and a trace of carbolic acid; fill the meatus full of this solution, and force it down through middle ear and Eustachian tube. I find that where the ear-drum is intact, and there is as yet no development behind it, acute otalgia can generally be relieved by very warm water, or warm solution of atropia sul., and that a timely operation for paracentesis of drum, when pus has accumulated, will hasten a cure and save the patient untold pain and suffering.

So the points I would make are—to use mild astringent remedies, combined with antiseptics, such as carbolic acid, boracic acid, etc., etc., thorough inspection of the parts, careful cleansing, and a special local treatment that has for its main object the destruction of these coccobacteria. One reason, in my mind, that bacteria are found in so many of these cases, is the fact that most cases applying are chronic ones, and have been accustomed for days at a time to plugging the meatus in order to get relief from the fetor, and protect their clothing.—[Chicago Medical Examiner.

